# Responses and Controlling Factors of the Litter Decay Rate to Nitrogen Addition Across Global Forests: A Meta-Analysis

**DOI:** 10.3390/plants14203221

**Published:** 2025-10-20

**Authors:** Lijun Fan, Weiwei Wang, Xuejun Liu, Yuan Su

**Affiliations:** 1 China West Normal University, Nanchong 637009, China; 2 Sichuan Provincial Engineering Laboratory of Monitoring and Control for Soil Erosion in Dry Valleys, School of Geographical Sciences, China West Normal University, Nanchong 637009, China; 3 Key Laboratory of Plant-Soil Interactions, College of Resources and Environmental Sciences, National Academy of Agriculture Green Development, Ministry of Education, National Observation and Research Station of Agriculture Green Development (Quzhou, Hebei), China Agricultural University, Beijing 100193, China

**Keywords:** common litter traits, common situ traits, decay rate, humidity index, litter quality, nitrogen addition, situ traits

## Abstract

Plant litter decay is an essential process for recycling C and nutrients in natural ecosystems. However, the impacts of N addition on litter decay are not well understood in global forest ecosystems. Therefore, a meta-analysis was used to examine how N addition affects the litter decay rate through three kinds of litter decay traits (i.e., common litter trait (litter collected from control plot is decomposed in N addition plots); common site trait (litter collected from N addition plots is decomposed in control plot); and in situ trait (litter collected from control and N addition plots is decomposed in situ)), based on 1145 field observations from 166 published studies on global forests. Nitrogen addition significantly reduced the litter decay rate by 3.92% across the three kinds of decay traits. However, there were different responses of the litter decay rate to N addition among the decay traits. The N addition notably inhibited litter decay by 5.99% for the common litter trait but consistently promoted it by 8.37% and 7.48% for common soil and situ traits, respectively. The magnitude and direction of such effects varied with the N addition amount, form and duration. The effect size of the litter decay rate due to N addition was negatively related to the initial N concentration and C:N ratio for the common litter trait. The N concentration in litter was raised by N addition, resulting in an increase in the litter decay rate for the common situ trait. For the situ trait, N addition increased N concentration and reduced C:N and lignin/N in litter, resulting in an increase in the decay rate, and the responses of the litter decay rate to N addition were also influenced by the humidity index. Overall, our results showed that the responses of the litter decay rate to N addition were different among the three kinds of decay traits and were controlled by environmental and experimental factors. These findings help us to better understand the effects of N addition on biogeochemical cycling in global forest ecosystems.

## 1. Introduction

Litter decay is pivotal in the primary supply of nutrients for plants in terrestrial ecosystems [1,2]. As well-proven in numerous studies, litter quality and soil decomposers regulate this process locally [3,4,5]. Nitrogen (N) addition significantly altered litter quality [6], soil microbe and enzyme activities [7,8,9], and soil fauna [10,11,12], and these changes would have a substantive impact on litter decay [13]. In the past century, human activity has significantly altered the Earth’s N cycle and accelerated reactive N emissions up to 92.7 Tg of N in 2020 [14]. Therefore, a comprehensive understanding of N addition effects on litter decay is vital for forecasting the influences of increments in N deposition on forest ecosystem structure and function.

Despite numerous studies of N effects on forest litter decay, the results vary greatly. Some studies found that N addition repressed the litter decay rate [15,16,17,18], while some reported negligible effects [16,19] or increases in the decay rate under N addition [20] in forest ecosystems. These contrasting responses of litter decay to N addition may result from differences in measuring decay traits. There are three common ways to examine the influence of N addition on litter decay [18]. Firstly, litter collected from the plots without N addition is decomposed into those with N addition (i.e., common litter trait), mainly used to explore N addition effects on litter decay via the impact on soil microbial characteristics or the soil environment [18]. Secondly, control plots are utilized to decompose litter acquired from N addition plots (i.e., common situ trait), which mainly examines the N addition effect on litter decay via effects on litter quality [18]. Thirdly, litter from each N addition plot was decomposed in the soil from the same treatment plot (also known as situ trait), which examines the effect of N addition on litter decay by means of the comprehensive effect on litter quality and soil microbial properties [18].

Litter decay is a complex procedure involving both biotic and abiotic processes [5]. On a global scale, there is a significant positive correlation between the rate of leaf litter and the annual mean temperature (MAT) as well as the annual mean precipitation (MAP) [21,22]. Therefore, MAP and MAT could influence the N effect on the litter decay rate. Studies have shown that N addition effects on soil microbial biomass, soil enzyme activity, and the composition of soil microbes and fauna are correlated with the amount, form, and duration of N addition [7,8,9,10]. Therefore, for the common litter trait, N addition is likely to exert variable effects on the litter decay rate with N addition amount [23], as well as the form and duration of N addition [24]. In addition, the decay rate is affected by N addition altering litter quality [18]. Generally, N addition enhances litter N concentrations while reducing litter C:N and lignin/N ratios [13,18], thereby potentially promoting decay because of the positive correlation between the litter decay rate and N and C:N in litter [22,25]. Therefore, we infer that N addition could promote litter decay due to improved litter quality for the common soil trait [18,26]. Nevertheless, the two traits may not truly reflect the effect of N addition on litter decay because it affected both litter quality and decomposers. Hence, the situ trait could be more realistic, which means that the N addition effects on litter decay are more complex and increases the uncertainties due to interactions among litter quality, soil nutrients, and decomposers [27,28]. The net effect of N on decay depends on the trade-offs among soil organism activity, soil nutrient conditions, and litter quality improvement [18,29]. Several meta-analyses have examined the effect of N addition on forest litter decay, showing both negative [23,24,25,30] and positive results [23,25], but they have not separated the three kinds of litter decay traits, increasing the uncertainty of the assessment results. Despite Wu et al. (2023) [13] reporting that N addition differently influenced the decay rate in the three kinds of litter decay traits, its effects on global forest litter decay rates remain unclear. Therefore, it is necessary to separate these three traits to evaluate N addition for its performance in influencing litter decay separately using a particular ecosystem.

The global forest area is approximately 4.06 billion hectares, accounting for 30.8% of the land area and representing a carbon stock of 870 ± 61 Pg C in 2020 [31]. It is highly sensitive to atmospheric N deposition, and even a slight change in the rate of organic matter decay could have a profound impact on global C balance and cycling [32]. Such ecosystems offer a unique platform to quantify the impact of N on litter decay because this has been examined in numerous studies using multiple N-level traits [15,16,19]. In this paper, we aimed to clarify the role of N addition on litter decay considering both direction (i.e., increase or decrease) and magnitude and to explore the factors influencing the litter decay rate in response to N addition among the three litter decay traits. We had the following three hypotheses: (I) N addition would inhibit the litter decay rate due to N-induced reductions in microbial biomass and activity in the common litter trait [7]; (II) N addition would promote the litter decay rate due to N-induced improvements in litter quality in the common soil trait [13]; and (III) N addition would have a minor impact on the litter decay rate in situ trait because it depends on the trade-off between the improvement of litter quality promoting decay and the decrease in microbial activity inhibiting decay. To test these hypotheses, a database of 166 publications including 1145 observations was compiled on litter decay in global forests. In addition, we focused on experimental (amount, form, duration of N addition, decay time, and litter quality) and environmental factors (MAT and MAP) affecting litter decay with N addition.

## 2. Results

### 2.1. N Addition Overall Effect on Litter Decay Rate

**Figure 1:**
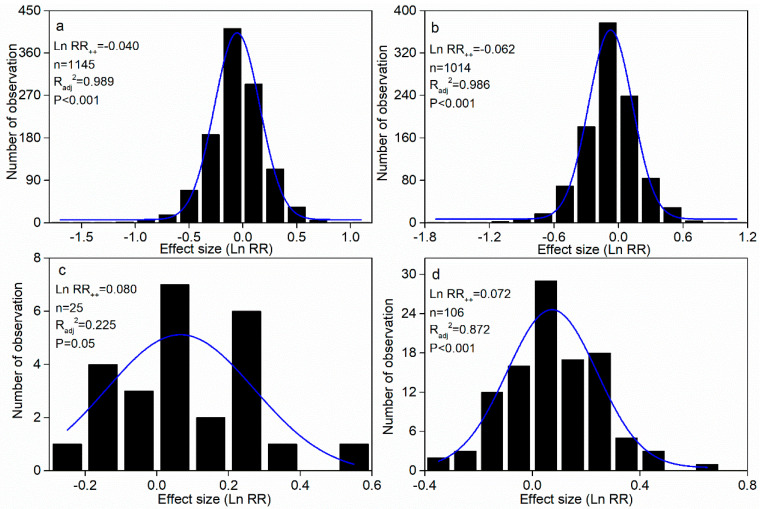
Frequency distribution of natural log response ratio (Ln RR) in total (**a**), common litter (**b**), common soil (**c**), and in situ (**d**) traits.

**Figure 2:**
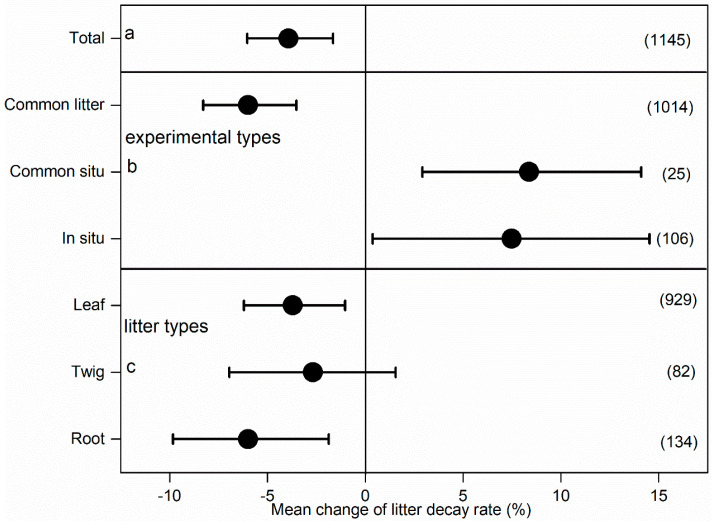
Influence of N addition on litter decay rate for different litter decay traits (means and 95% CIs are shown).

**Figure 3:**
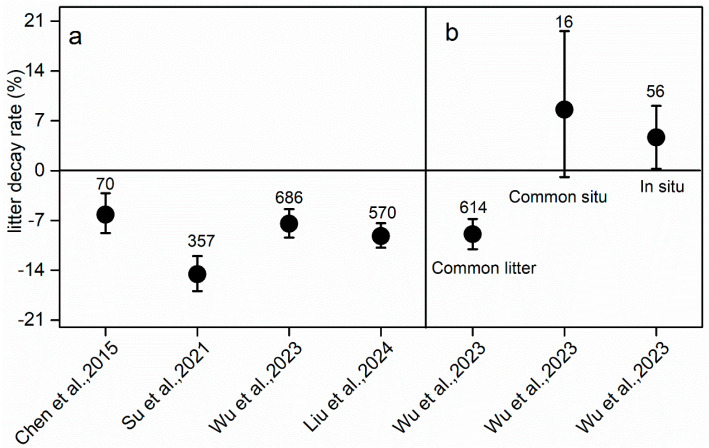
Comparison of the impact of N on the litter decay rate among previous meta-analyses in forest ecosystems. The sample sizes are given above the bars.

All data in this meta-analysis met the normality criteria (Figure 1a–d). The N addition significantly reduced the rate of global forest litter decay by 3.92% [the data showed positive (434), neutral (14), and negative (697) effects], with 60% of the datasets showing a negative effect on the decay rate (Figure 1a and Figure 2). Specifically, N addition for the common litter trait markedly slowed the decay rate by 5.99% (95% CI: 2.06–2.48%, n = 1014) (Figure 1b and Figure 2). Similarly, for common situ and situ traits, N addition markedly promoted the litter decay rate by 8.37% (95% CI: 5.45–5.75%, n = 25) and 7.48% (95% CI: 7.06–7.11%, n = 105), respectively (Figure 1c,d and Figure 2). The N addition also had different effects on leaf, twig, and root decay rates, with negative effects on leaves and roots and neutral effects on twigs. In addition, comparing the results with the previous meta-analysis, N addition consistently had a negative impact on litter decay across all trait types and for the common litter decay trait but tended to promote litter decay for common soil and situ traits (Figure 3).

### 2.2. Amount, Form, and Duration of N Addition Effect on Decay Rate

**Figure 4:**
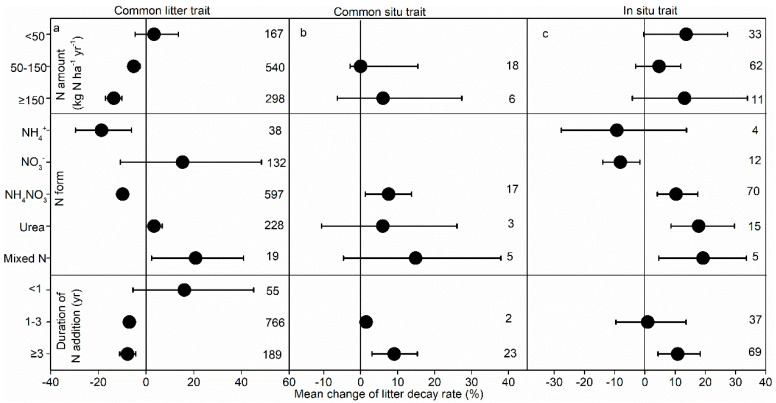
Influence of amount, form, and duration of N addition on litter decay rate.

For the common litter trait, increasing the N addition amount inhibited the litter decay rate (Figure 4a). However, raising the N addition amount did not markedly influence the decay rate for the common soil and situ traits (Figure 4b,c). The form of N addition significantly influenced N effects on litter decay. Specifically, NH_4_^+^ and NH_4_NO_3_ addition notably reduced the litter decay rate, but mixed N addition consistently promoted decay for the common litter trait (Figure 4a). For common soil and situ traits, NH_4_NO_3_ addition significantly enhanced the litter decay rate (Figure 4b,c); urea and mixed N addition notably enhanced the litter decay rate for the situ trait but did not significantly affect decay for the common soil trait. Also, the duration of N addition affected the litter decay rate (Figure 4); N addition for more than 1 yr increased the litter decay rate for the common litter trait, but long-term N addition (≥3 yr) consistently decreased decay in common soil and situ traits (Figure 4b,c).

### 2.3. Decay Time, Mesh Size, and Litter Type Influence N Effects on Litter Decay

**Figure 5:**
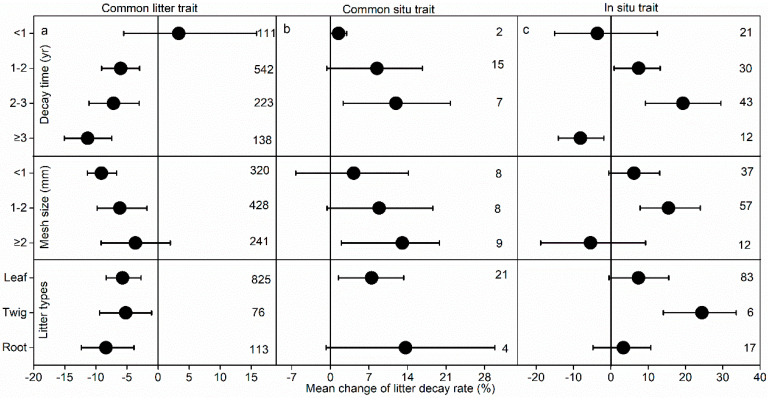
The influence of N on the litter decay rate as related to decay time, mesh size of the litterbag, and litter type.

The decay time significantly influenced the effect size on the litter decay rate of N addition (Figure 5). Short-term N addition (<1 yr) did not markedly influence litter decay for the three kinds of traits (Figure 5). Specifically, the negative effect of N influencing the decay rate was stronger for decay time ≥1 yr in the common litter trait (Figure 5a). N addition markedly enhanced the litter decay rate for a decay time of 2–3 yr in common soil and situ traits (Figure 5b,c) but notably decreased decay for the in situ trait with decay time ≥3 yr.

The mesh size of litterbags also influenced the N addition effect on decay (Figure 5). The N addition facilitated litter decay in litterbags of small size (<2 mm) only for the common litter trait but increased litter decay for litterbags of large size (≥2 mm) and 1–2 mm for common soil and in situ traits, respectively. In addition, litter type also affected the decay rate for the three traits. N addition consistently reduced the decay rate of leaves, twigs, and roots for the common litter trait (Figure 5a). N addition notably enhanced the leaf decay rate for the common soil trait (Figure 5b) and the twig decay rate for the situ trait (Figure 5c).

### 2.4. Humidity Index and Litter Quality Influence N Effects on Litter Decay

**Figure 6:**
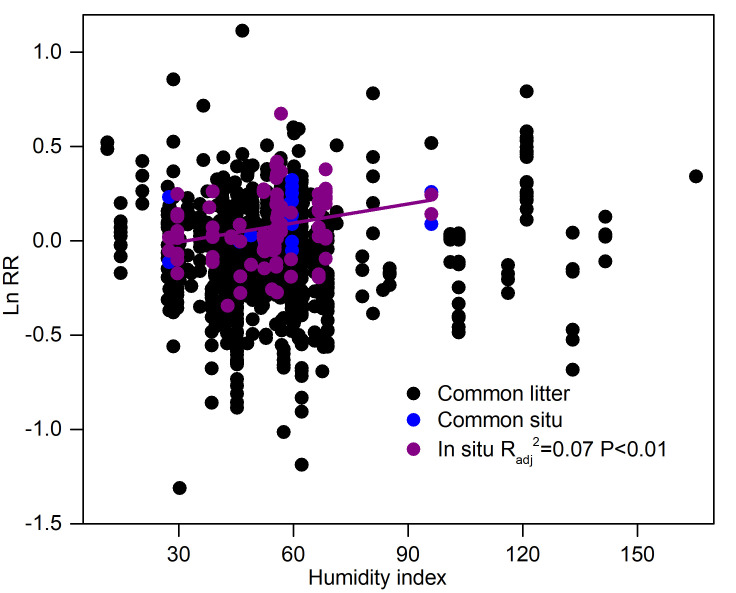
The influence of N on the litter decay rate as related to the humidity index.

**Figure 7:**
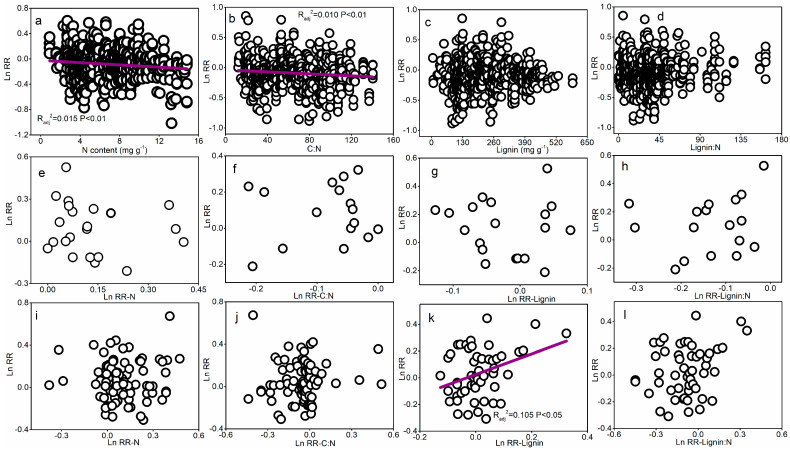
The influence of N on the litter decay rate as related to litter quality for common litter (**a**–**d**), common situ (**e**–**h**), and in situ (**i**–**l**) traits.

The MAP, MAT, and humidity index altered the effect size of the litter decay rate in regard to N addition (Figure S3 and Figure 6). For the situ trait, there was a positive relationship between the humidity index and the effect size of the decay rate, but effect size did not show any significant correlations with the humidity index in common litter and soil traits (Figure 6). N addition significantly enhanced litter N concentration, reduced C:N and lignin/N ratios (Figure S4), and influenced N effects on the decay rate (Figure 7). Specifically, as litter N concentration and the C:N ratio increased, the Ln RR decreased linearly for the common litter trait (Figure 7a,b). There was no marked correlation between Ln RR and lignin concentration and lignin/N (Figure 7c,d). Conversely, there was a positive relationship between the Ln RR–lignin and Ln RR–lignin/N and the Ln RR of the decay rate for the situ trait (Figure 7k,l) but not for the common soil trait (Figure 7g,h). There was no significant relationship between Ln RR and Ln RR-N (or LnRR-C:N) for common soil and situ traits (Figure 7e,f,i,j).

## 3. Discussion

Our meta-analysis investigated the impact of N addition on the litter decay rate using three kinds of litter decay traits in global forests. Across the three kinds of litter decay traits, N addition notably slowed the litter decay rate by 3.92%, consistent with previous meta-analysis about N influencing litter decay in regional and global forests [13,24,25]. In addition, our results clearly demonstrated that N addition differently influenced the litter decay rate among three kinds of traits. Specifically, N addition markedly slowed the litter decay rate by 5.99% for the common litter trait but markedly accelerated the litter decay rate by 8.37% and 7.48% for the common situ and situ traits, respectively (Figure 2b). These findings provide a comprehensive understanding of the impacts of N addition on the decay rate of global forest litter.

### 3.1. N Influencing Litter Decay Rate in Common Litter Trait

The litter decay rate was significantly reduced by N addition for the common litter trait (Figure 1 and Figure 2), supporting our first hypothesis. However, N addition did not always promote the decay rate for the common litter trait. Specifically, low N addition (<50 kg ha^−1^ yr^−1^) had a minimal effect on the litter decay rate (Figure 3a), and the low sensitivity of the litter decay rate under the condition of low N addition might be related to the insignificant influence of low N addition on the activity of soil decomposers [12]. However, medium and higher amounts significantly inhibited the litter decay rate, with more negative effects with N addition amounts, which might result from declines in abundance of microbes, which were more evident in studies with higher total amounts of N added [33]. N-induced soil acidification resulted in base cation loss, further leading to restricted microbial base cations as well as increased toxicity to microbes from manganese and aluminum ions (Mn^2+^ and Al^3+^) [34,35] and, finally, a decreased soil microbial biomass and decay rate.

The form and duration of N addition had marked impacts on the litter decay rate (Figure 3a). The addition of NH_4_NO_3_ and NH_4_^+^ consistently decreased the decay rate, while mixed N and urea addition increased it, partly consistent with the meta-analysis by Wu et al. (2023) [13]. Mixed N addition consistently enhanced soil hydrolase and oxidase activities [9], which might increase the litter decay rate. In most cases, soil enzyme activity is not equivalent to soil microbial activity, nor does it reflect the nutrient cycle [36]. The addition of N in mixed forms provided a wider N source growth range for microorganism decay [37], therefore enhancing the decay rate. Additionally, the inhibitory effects became stronger with an increasing duration of N addition and decay time, consistent with previous studies [13,25] because long-term N addition represses lignin-degrading metabolism and reduces litter lignin decay at the later stages [9,38,39], emphasizing the importance of investigating long-term decay traits.

The effect size of the litter decay rate was negatively correlated with initial N and C:N ratios for the common litter trait (Figure 7), which suggested that N addition may have stronger inhibitory effects for high-quality litter. The findings did not support the results of a previous study showing that N addition could stimulate the decay of high-quality litter but inhibit that of low-quality litter from forest ecosystems [38], which is possibly associated with the “microbial N mining” hypothesis [40]. This hypothesis states that some microbes decompose recalcitrant organic matter by virtue of labile C, thereby producing N. The addition of external N met the need for N of microbes and, subsequently, the resources required by the microbes for the decay of recalcitrant organic components would decrease. Under such a condition, it is estimated that N addition would have stronger negative effect on decay, especially for high-quality litter [40]. Zhang et al. (2016) [17] also found that N addition has a more significant negative impact on the decay of high-quality litter. However, our research did not find any correlation between the effect size of the decay rate and initial lignin content and the lignin/N ratio.

### 3.2. Influence of N on Litter Decay Rate in Common Situ and Situ Traits

For the common situ trait, N addition markedly increased the litter decay rate (Figure 2, n = 25), supporting our second hypothesis. We performed a thorough reanalysis of the previous results of Wu et al. (2023) [13], and this showed that N addition tended to promote the decay of forest litter for the common soil trait but did not reach a significant level, which was not consistent with our results. This difference might result from inconsistent sample sizes (Figure 3, n = 16). The increased litter decay rate resulted from a better litter quality owing to N addition [13]. Concentrations of litter N climbed with N addition, whereas C:N and lignin/N significantly decreased, so N addition improved litter quality (Figure S2). Generally, litter with high N levels shows more rapid decay than litter with low nutrient contents due to the stimulation of decomposer growth and activity from the high-quality litter [41,42]. However, improved litter quality does not always promote litter decay for the common soil trait [18], because it has been demonstrated that available nutrients in litter [e.g., calcium (Ca) and Mn, except for N] can influence the litter decay rate [43]. Both Ca and Mn are necessary for microorganism metabolism [44] and saprotrophic fungi also require these for generating lignolytic enzymes used in lignin decay [44]. However, due to the limited data, we cannot provide much discussion concerning the common soil decay trait.

Interestingly, N addition notably increased the litter decay rate (in situ trait, n = 106 observations from 24 studies) (Figure 2), which did not support our third hypothesis. For the situ trait, significant amelioration of litter quality was also detected (Figure S2), consistent with a previous meta-analysis showing that N addition markedly enhanced the litter decay rate by 4.66% [13]. Nonetheless, there is a lack of information on N addition affecting soil microbial-related data for situ litter decay traits, because only 6 of the 24 studies reported microbiome-related data, and the detection indicators and methods were inconsistent [18,45,46,47,48]. For example, Wang et al. (2024) found that N addition significantly decreased numbers of soil fauna across all sampling periods, which might have reduced the decay process involving soil fauna [49]. Additionally, N addition reduced lignolytic enzyme activity [18], and these changes would influence the decay process. Liu et al. (2010) [29] discovered that N addition raised the N concentration in litter and hence promoted litter decay through increasing substrate quality. Nevertheless, such an increase was counterbalanced by raised soil N content via its negative effect on soil microbial biomass and activity, so that the enhanced soil N availability had a diminished impact on in situ litter decay. The increased litter quality had positive effects that slightly exceeded the negative impacts of raised soil N, finally causing increased decay. However, Wei et al. (2022) [50] found that for all species and their mixture, the litter decay rate declined persistently with N addition in the situ decay trait in an alpine steppe. Although N addition increased the litter quality, the decreasing decay rate with higher N addition was attributable to attenuation of soil bacterial diversity and of UV radiation. Similarly, van Diepen et al. (2015) [18] found that high internal N, in combination with low Ca and Mn levels, exerted an inhibitory effect on lignolytic enzyme activity, further reinforcing the N-triggered repression for the situ decay trait. The above results suggested that the interactions of N addition, litter quality, and enzyme activity jointly influenced litter decay. Therefore, for the situ decay trait, the influence of N on the litter decay rate depends on the trade-off between the positive impact of N addition on decay due to improvements in litter quality and the negative impact on decay caused by reduced soil biological activity.

The effect size of the litter decay rate to N addition is positively correlated with the effect size of lignin concentration and lignin/N to N addition in the situ decay trait, which also further demonstrated that litter quality plays an important role in influencing the litter decay rate [21,22]. In addition, the humidity index was positively associated with the effect size of the litter decay rate regarding its response to N addition (Figure 6), indicating that the increase in the humidity index magnifies the positive effect of N addition on the litter decay rate. In moisture-limited ecosystems, particularly arid and semi-arid biomes, water constraints may supersede N controls in determining the decay rate due to the critical role of water in mediating microbial metabolic processes [5,51]. Our study suggested that the interaction between water and N plays a vital role in controlling the decay of global forest litter.

### 3.3. Limitation and Future Studies

Although our meta-analysis showed the influence of N addition on the decay rate of litter across global forest biomes, persistent uncertainties stem from fragmented data of the interactions among the decay rate, substrate quality, and soil biological factors. Firstly, most studies are mainly derived from forest ecosystems in the mid-lower latitudes of the Northern Hemisphere. The current findings require further verification in high-latitude ecosystems of the Northern Hemisphere (e.g., boreal forests or tundra), where distinct climatic and edaphic conditions may alter decay dynamics. Secondly, the compiled dataset included relatively few studies with the duration of N addition and decay exceeding 3 yr, meaning that the stage of complete litter decay was not reached [1,52,53]. Thirdly, despite rigorous data curation efforts, there were limited sample sizes for common soil and situ decay traits and special organs (e.g., root and stem litter). Therefore, it is strongly recommended that future studies should consider in situ decay over longer periods, particularly in the development of more intricate dual-pool models, and further analyses should combine the soil community composition and consider litter substrates of different organs, which are the most important contributors to variation in the decay rate, which will allow for the disentanglement of the mechanisms of the decay process. Furthermore, fine roots and stems in forests represent 11% and 48% of annual litter, respectively [54]. In addition, different factors influence the decay of root, stem, and leaf litter [42], so research on underground parts and stem litter decay should be strengthened. Other than N, it has been corroborated that availability of other nutrients (e.g., Ca, magnesium, and Mn) can influence litter decay [22,43], while N addition markedly affects litter macro- and micro-nutrients of metal ions in forest ecosystems [55,56,57]. Therefore, the influence of changes in these elements on litter decay should be investigated.

## 4. Materials and Methods

### 4.1. Collection of Data

The datasets from the China National Knowledge Infrastructure and Web of Science were retrieved using the following keywords to obtain 1145 observations plus 166 publications (Figure S1) (published from January 2000 to March 2025): “N fertilization” OR “N enrichment” OR “N input” OR “N deposition” OR “N addition” and “litter decomposition” OR “litter decay” OR “mass loss”. To avoid bias, we selected the articles using the following criteria: (1) the litterbag method was used to determine the litter decay trait; (2) data collection was conducted only on field N addition traits; (3) the single-pool exponential decay model [58] was utilized for the calculation of the decay rate (k value); (4) if investigations did not directly report the decay rate, reports were made on the percentage remaining or loss of litter mass at different time points (three at least) in the whole period—we used the single-pool exponential decay model to calculate the decay rate; and (5) only treatments with N addition and a control were chosen to acquire data for multifactorial studies for the purpose of preventing other factors from influencing the interaction. Web Plot Digitizer was employed to directly obtain or extract data from tables or graphs, respectively.

Geographical information [latitude and longitude (Figure S2), MAT and MAP], initial litter quality [N, C:N, lignin, and lignin/N], litter type (leaf, twig, and root) and trait type (common litter, common soil, and in situ) were recorded. The duration of N addition (<1, 1–3, and ≥3 yr), decay time (<1, 1–2, 2–3, and ≥3 yr), litterbag mesh size (<1, 1–2, and ≥2 mm), N addition form [ammonium (NH_4_^+^), nitrate (NO_3_^−^), ammonium nitrate (NH_4_NO_3_), urea and mixed N (including organic and inorganic N added)], and amount (<50, 50–150, and ≥150 kg N ha^−1^ yr^−1^) were grouped mainly based on the work by Wu et al. (2023) [13] and Liu et al. (2024) [25].

### 4.2. Meta-Analysis

The N influence on the decay rate was examined using effect size determined as the natural log of the response ratio (RR) [59]:

(1) $$\ln\left(RR\right)=\ln\left(\frac{k_{t}}{k_{c}}\right)$$

where k_t_ and k_c_ correspond to the decay rate from N addition and without N addition treatments.

The sample size in each trait was selected for calculating a weighting factor (w) [60,61]:

(2) $$w=\frac{{n_{c}*n}_{t}}{{n_{c}+n}_{t}}$$

where n_c_ and n_t_ are the sample repetitions for N addition and no N addition treatments, respectively.

If a study contributed multiple observations to the analysis, we proportionally adjusted the weighting factors based on the site-specific observation. The final weight (w′) was used [62]:

(3) $$w^{\prime }=\frac{1}{w*n}$$

where n is the number of observations from one study.

The calculation of ln $\left({RR}^{\prime }\right)$ as the weighted effect size is as follows:

(4) $$ln{RR}^{\prime }=w^{\prime }\times \ln\left(RR\right)$$

Finally, the formula below was applied to clarify how N addition influences the decay rate through the weighted effect size (lnRR_++_):

(5) $$\left(ln{RR}_{++}\right)=\frac{\sum {ln{RR}^{\prime }}_{i}}{\sum {w^{\prime }}_{i}}$$

where ${ln{RR}^{\prime }}_{i}$ and ${w^{\prime }}_{i}$ represent the $ln{RR}^{\prime }$ and $w^{\prime }$ of the ith observation, respectively.

MetaWin 3.0 was used for calculating the final weighted RR and 95% bootstrap confidence interval (CI) [63]. We used a random-effect model to establish all weighted RR calculations as well as categorical comparisons via MetaWin 3.0. A total of 9999 iterations were conducted to obtain the 95% bootstrap CI. If the condition of 95% bootstrap CI values failed to overlap zero, it was deemed as significant. Given the response of the litter decay rate to N addition relating to experimental and environmental factors (Wu et al., 2023) [13], we adopted a categorical random-effect model to examine the mean effect size of different groups among the environmental and experimental factors, and categorical groups were deemed to be significant differences when the 95% CI did not overlap with each other (Wu et al., 2023) [13]. In order to facilitate comparison, N-induced percentage change was calculated:

(6) $$change(\%)=\left(e^{ln{RR}_{++}}-1\right)\times 100$$

We used a Gaussian function to reflect variability among individual studies with the following function [64]:

(7) $$y=\alpha {exp}^{-\frac{{\left(x-\mu \right)}^{2}}{2\sigma^{2}}}$$

where x and y show the average RR and corresponding frequency in a single interval, respectively; α is the estimated RR value at x = µ; µ indicates the mean of the ln RR normal distribution; and σ^2^ denotes the corresponding variance.

Regression analyses were also used to determine the correlations between the effect size of the decay rate, environmental factors [MAP, MAT, and $humidityindex=(\frac{MAP}{MAT+10})$ [24]], and the effect size of litter quality.

## 5. Conclusions

Our meta-analysis clearly showed different responses of the litter decay rate to N addition among three kinds of litter decay traits in global forest ecosystems. Nitrogen addition significantly reduced the litter decay rate for the common litter trait but promoted it in common situ and situ traits. Therefore, studies that only considered the effect of N addition on the litter decay rate via its effect on the soil environment or microbial properties and via its effect on litter quality might not be suitable to accurately assess the promoting effect of N on litter decay. The negative effect on the decay rate became stronger with longer durations and amounts of N addition for the common litter trait, while the positive effect on the decay rate became stronger with longer durations of N addition for the common litter and situ traits when decay time was <3 yr; however, the shift from promotion to inhibition occurred when the decay time was ≥3 yr only for the situ decay trait. In addition, the increase in the humidity index significantly amplified the positive impact of N on litter decay only for the situ trait. These findings suggested that experimental (N form, litter decay time, duration, and litter quality) and environmental (MAT, MAP) factors also regulated the effect of N on litter decay. However, due to data limitations for the situ trait, we were unable to analyze the mechanism of N addition by taking advantage of its comprehensive impacts on litter soil microbial properties to influence litter decay. Therefore, long-term situ decay traits are required to accurately evaluate litter decay in response to increasing N deposition.

## Data Availability

The original contributions presented in this study are included in the article/Supplementary Material. Further inquiries can be directed to the corresponding author.

## Supplementary Materials

The following supporting information can be downloaded at: https://www.mdpi.com/article/10.3390/plants14203221/s1, Figure S1. PRISMA 2020 standards for study selection in this meta-analysis. Figure S2. Distribution of N addition traits included in this meta-analysis in global forests. Figure S3. N addition effect on litter quality in common soil traits and in situ decay trait. Figure S4. Relationships between effect size of litter decay rate (ln RR) and MAT (a) and MAP (B) in the three kinds of decay trait.
