# Supplementary material for: Responses and Controlling Factors of the Litter Decay Rate to Nitrogen Addition Across Global Forests: A Meta-Analysis
Source: Plants (Basel). 2025 Oct 20;14(20):3221. doi: 10.3390/plants14203221 (PMC12566841; doi:10.3390/plants14203221)
Supplement: Supplementary file 1 [file plants-14-03221-s001.zip › plants-3896764-supplementary.pdf]

# Supporting information for

Responses and controlling factors of litter decay rate to nitrogen addition across global forests: A meta-analysis

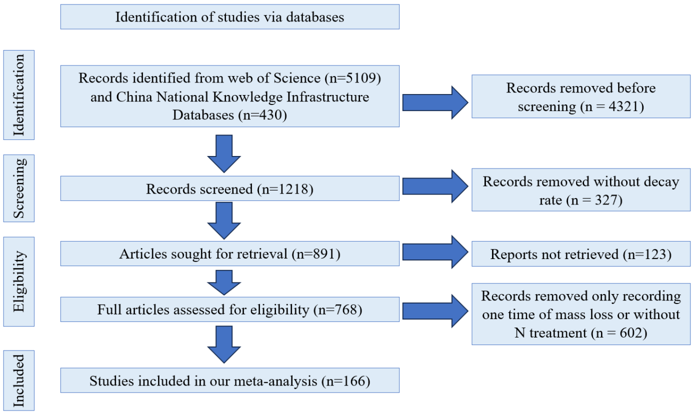


**Figure S1** PRISMA 2020 standards for study selection in this meta-analysis.

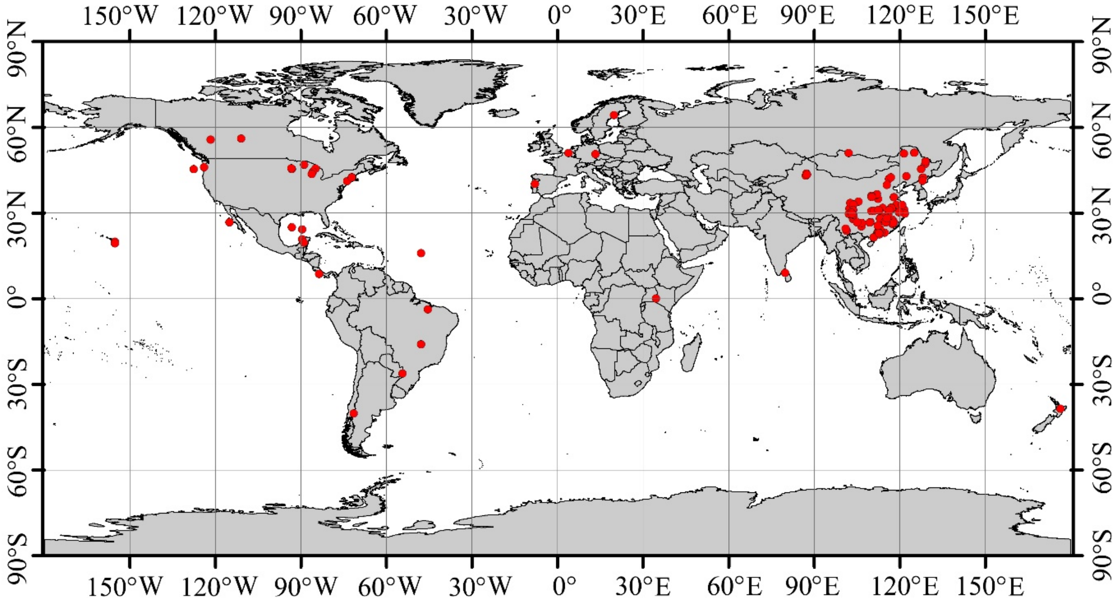


**Figure S2** Distribution of N addition traits included in this meta-analysis in Chinese grasslands.

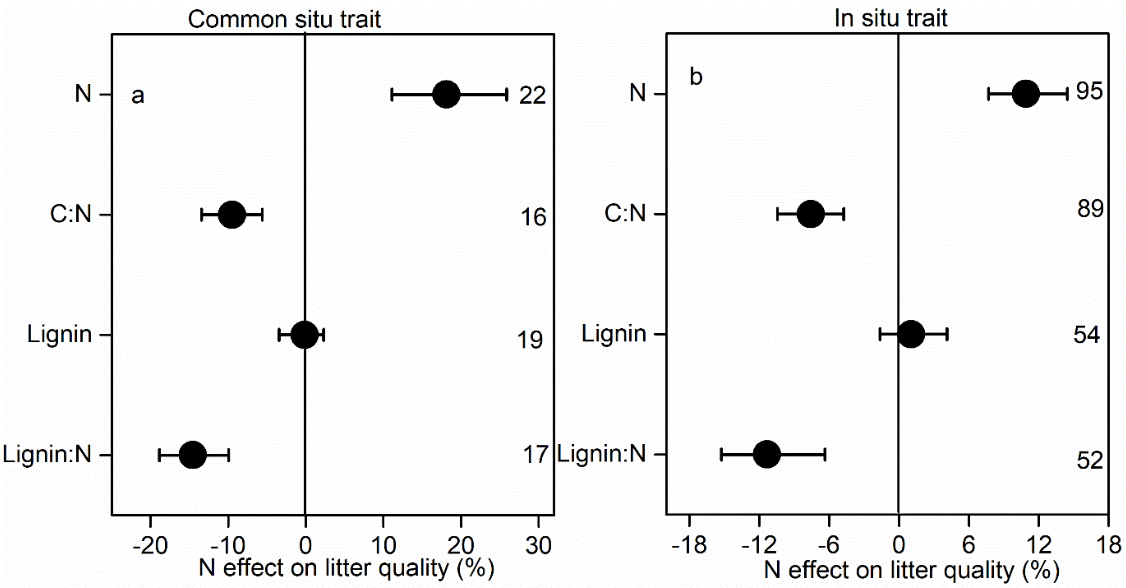


**Figure S3** N addition effect on litter quality in common soil traits and in situ decay trait.

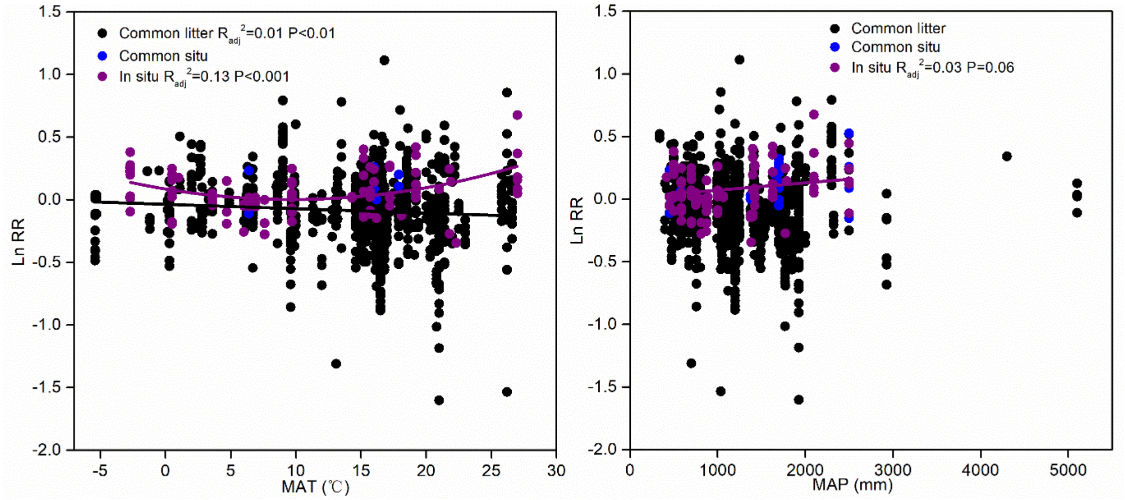


**Figure S4** Relationships between effect size of litter decay rate (ln RR) and MAT (a) and MAP (B) in the three kinds of decay trait.
